# Irisin attenuates cardiac injury and improves prognosis in rats with hemorrhagic shock by maintaining mitochondrial homeostasis via the AMPK/Drp1 pathway

**DOI:** 10.3389/fphar.2025.1560608

**Published:** 2025-04-28

**Authors:** Zheng Zhang, Yufang Zhang, Xiaofang Zou, Jiake Li, Yunfei Chi, Hailiang Bai, Bin Wei, Huiting Yun, Quanxi Zhang, Weihua Cao, Haiyan Liu, Hongjie Duan

**Affiliations:** ^1^ Department of Diagnosis and Treatment for Cadre, Fourth Medical Center, Chinese PLA General Hospital, Beijing, China; ^2^ Graduate School, Hebei North University, Zhangjiakou, China; ^3^ Department of Burns and Plastic Surgery, Peoples Liberation Army Air Force General Hospital, Beijing, China; ^4^ Basic Medical College, Shanxi Medical University, Taiyuan, China

**Keywords:** irisin, hemorrhagic shock, mitochondrial homeostasis, AMPK/Drp1 pathway, cardiac injury

## Abstract

**Objective:**

Hemorrhagic shock (HS) is a critical clinical condition in which cardiac dysfunction and failure are leading causes of mortality. Mitochondrial dysfunction is central to the pathogenesis of cardiac dysfunction in HS. Irisin has been shown to improve mitochondrial function and protect against ischemia-reperfusion injury (IRI), but its specific effects on myocardial injury in HS are unknown. This study investigates irisin’s therapeutic potential in a rat model of HS.

**Methods:**

For *in vivo* studies, a rat HS model was established via controlled blood withdrawal and Animals were allocated to four groups: Sham, HS, HS + Vehicle (HS + Veh), and HS + Irisin. Physiological responses were evaluated through temporal sampling at 1, 3, and 6 h post-HS. For *in vitro* studies, H9c2 cardiomyocytes were exposed to oxygen-glucose deprivation to establish a hypoxic model. Cells were categorized into six groups: normoxia (N), normoxia + AMPK inhibitor compound C (N + Cc), hypoxia (H), hypoxia + Cc (H + Cc), hypoxia + irisin (H + Irisin), and hypoxia + Cc + irisin (H + Cc + Irisin). Cellular functional outcomes were analyzed following 3-h hypoxia exposure.

**Results:**

HS significantly reduced serum irisin levels. Exogenous irisin administration enhanced survival rates, stabilized mean arterial pressure (MAP), lowered lactate (LAC) levels, improved cardiac structure and function, and reduced myocardial injury biomarkers in HS rats. Mechanistically, irisin activated AMP-activated protein kinase (AMPK) and Sirtuin 1(SIRT1), to suppress the expression of dynamin-related protein 1 (Drp1) and fission protein 1 (Fis1), while upregulating mitofusin 1 (Mfn1). This modulation of mitochondrial dynamics preserved cardiomyocyte mitochondrial membrane potential (MMP), ATP production, and structural integrity. Hypoxic H9c2 cardiomyocytes exhibited consistent results. To confirm AMPK/Drp1-dependent mechanisms, Cc was administered to inhibit irisin-induced AMPK activation. Cc abolished irisin’s suppression of Drp1/Fis1 and its Mfn1 upregulation. Furthermore, Cc eliminated irisin-mediated protection in both H9c2 cardiomyocytes and mitochondria.

**Conclusion:**

Our study demonstrates that irisin ameliorates cardiac function and enhances early prognosis in HS. These cardioprotective effects are achieved through attenuation of myocardial damage and SIRT1/AMPK/Drp1 pathway-dependent restoration of mitochondrial homeostasis.

## Introduction

HS, often caused by trauma that leads to rapid and substantial blood loss beyond physiological compensatory capacity, remains a leading cause of mortality among individuals under 44 years of age, accounting for 30%–40% of deaths, with an estimated 1.5 million fatalities annually worldwide (Han et al., 2022; Hogarty et al., 2023). The pathophysiology of HS is characterized by severe circulatory collapse and hypovolemia, which trigger a cascade of events, including inadequate tissue perfusion, neuroendocrine stress responses, and activation of the coagulation system, potentially culminating in metabolic derangement, organ dysfunction, and sustained hemodynamic instability (Zhang et al., 2024; Faria et al., 2022). The development of therapeutic strategies for early intervention to protect organ function in HS is therefore urgently needed. A significant body of research has identified cardiac dysfunction and failure as the primary causes of death in HS patients (Rushing and Britt, 2008). Myocardial cells are rich in mitochondria, which play crucial roles in sustaining cardiac function. Insufficient blood supply disrupts mitochondrial quality control mechanisms, leading to mitochondrial dysfunction. This dysfunction directly triggers cardiomyocyte apoptosis or necrosis, significantly contributing to cardiac injury and functional impairment (Du et al., 2022). Consequently, future research should focus on protecting myocardial mitochondria to identify novel therapeutic targets and methodologies for HS, enhance early treatment efficacy, and improve patient outcomes.

As essential cellular organelles, mitochondria undergo continuous cycles of fission and fusion, maintaining their highly dynamic nature. This process is crucial for maintaining cellular redox balance and energy homeostasis, thereby regulating cellular function (Tábara et al., 2024; Hsu et al., 2021). The protein Drp1 and its binding partner Fis1 are primarily responsible for regulating mitochondrial fission. Cytosolic Drp1 is actively recruited to mitochondria, binds to Fis1 on the outer mitochondrial membrane (OMM), and assembles into oligomers. These oligomers constrict and sever the mitochondrial membrane through a GTP-dependent contraction mechanism, ultimately driving mitochondrial fission (Du et al., 2022; Yu et al., 2021). Concurrently, Mfn1 mediates mitochondrial fusion (Hsu et al., 2021; Vongsfak et al., 2021). The literature increasingly suggests that excessive mitochondrial fission and reduced fusion can worsen mitochondrial dysfunction, leading to decreased ATP production and reduced MMP, which results in insufficient energy supply and subsequently causes systemic circulatory collapse, multiorgan failure, and increased lethality (Du et al., 2022; Liu et al., 2024; Lei et al., 2020). Studies have shown that Mdivi-1, a mitochondrial fission inhibitor, protects against circulatory and organ dysfunction in HS rats by inhibiting Drp1 translocation to mitochondria and fission (Hu et al., 2024). Therefore, modulating mitochondrial dynamics represents a promising therapeutic strategy for preventing and treating organ damage.

AMPK, a highly conserved serine/threonine kinase, is activated under metabolic stress, such as hypoxia, glucose deprivation, or elevated AMP/ATP ratios (Dengler, 2020). Research has demonstrated that during HS, AMPK expression in cardiac tissue increases as a defensive mechanism against metabolic disarray (Vongsfak et al., 2021). Moreover, its activation may also involve SIRT1, an evolutionarily conserved NAD^+^-dependent deacetylase (Lovy et al., 2020). Research has shown that AMPK and SIRT1 interact, forming a core regulatory network for energy metabolism and regulating mitochondrial dynamics (Fulco et al., 2008; Cantó et al., 2009; Hou et al., 2008). In addition, AMPK is a crucial regulator of mitochondrial dynamics. Phosphorylation of AMPK reduces Drp1-Fis1 binding to the OMM and enhances Mfn1 expression, thereby inhibiting mitochondrial fission and promoting fusion, which is protective against ischemic heart disease (Du et al., 2022), which may be associated with the deacetylation function of SIRT1 (Lovy et al., 2020; Song et al., 2021). However, the specific mechanisms underlying the roles of SIRT1 and AMPK in mitochondrial fusion and fission in HS-induced cardiac injury require further investigation.

Irisin, a recently identified myokine dependent on peroxisome proliferator-activated receptor-γ coactivator-1α, is abundantly expressed in muscle and cardiac tissues and is secreted into the circulation by tissue cells to modulate energy metabolism during physical activity (Boström et al., 2012; Li et al., 2015). Moreover, irisin modulates cardiac function by influencing cardiovascular regulatory neurons within the central nervous system, suggesting its potential as an effective cardiac function modulator (Brailoiu et al., 2015). However, the role and significance of irisin in modulating cardiac dysfunction induced by HS remain unknown. Given that AMPK is a key regulator of the cellular energy balance and plays a pivotal role in cellular growth and metabolism (Herzig and Shaw, 2018; Jia et al., 2024), we hypothesize a potential link between irisin and AMPK signalling. Studies have shown that upregulating Irisin improves cardiac function and inhibits myocardial fibrosis in rats with myocardial infarction via the AMPK/SIRT1 pathway (Li et al., 2024). Additionally, activating AMPK prevents mitochondrial dysfunction and cardiac IRI in diabetic mice (Xin et al., 2020). Furthermore, irisin mitigates IRI-induced oxidative stress and apoptosis in hepatic tissue by suppressing excessive mitochondrial fission, promoting mitochondrial fusion, and enhancing biogenesis (Bi et al., 2019). Therefore, this study aims to investigate whether irisin can ameliorate the mitochondrial dysfunction induced by HS, reduce mitochondrial fission, and thereby protect cardiac function and improve survival rates of HS rats via the SIRT1/AMPK/Drp1 pathway. This research may offer valuable insights for the development of innovative therapeutic strategies.

## Materials and methods

### Nonlethal HS animal model

Male Sprague‒Dawley rats (6–7 weeks old; 240–260 g) were obtained from Beijing Keyu Breeding Center (China). Male rats were selected to avoid estrogenic interference with HS responses (Soliman, 2015). The protocol commenced with intraperitoneal administration of sodium pentobarbital (50 mg/kg initial dose) for anesthesia. After the experiment, rats were euthanized by intraperitoneal injection of a lethal dose of pentobarbital sodium (100 mg/kg) to ensure a painless death. Cervical dislocation was performed after death as a secondary confirmation. Under sterile conditions, arterial and venous catheters were inserted into the left carotid artery and right jugular vein, respectively. To prevent clotting, systemic heparinization was performed through sodium heparin injection (100 U/kg). After 10 min of stabilization, 40% of the total blood volume (estimated as 6% body weight + 0.77 mL) was withdrawn through the carotid artery in 20 min to simulate clinically treatable HS cases in clinical settings (Bruhn et al., 2018). Blood withdrawal was conducted in two phases: 20% within 7 min, followed by the remaining 20% over 13 min. Rats surviving 30 min post-HS were considered to have successful HS induction.

Rats were randomly divided into four experimental groups (n = 8 per group):

1. Sham group: Underwent catheterization without blood withdrawal or treatment.
2. HS group: Underwent 40% total blood volume withdrawal without further intervention.
3. HS + Veh group: Following 40% blood withdrawal, rats received 0.5 mL lactated Ringer’s solution (vehicle control) through the right jugular vein.
4. HS + irisin group: Post-HS, rats received 250 µg/kg irisin (dissolved in 0.5 mL lactated Ringer’s solution; Kanglang, China) through intravenous infusion. The dose was selected based on preliminary experiments and prior literature (Liu et al., 2023).

### Establishment of a fatal HS model

The lethal HS model replicated the nonlethal protocol but induced 50% total blood loss (25% withdrawn within 7 min; remaining 25% over 13 min). This model is crucial for evaluating survival rates and irisin efficacy in extreme conditions, simulating severe clinical HS cases akin to patients dying from excessive bleeding without timely treatment (Hao et al., 2024). Following shock induction, survival was monitored for 72 h (n = 10 per group).

### Hypoxic cell model

H9c2 cardiomyocytes (Servicebio, China) were cultured in complete medium under standard conditions (37°C, 5% CO_2_, 100% humidity; n = 8 per group). For hypoxia induction, cells were transferred to serum/glucose-free medium (Seven/Abcells, China) and exposed to hypoxic conditions (37°C, 94% N_2_, 5% CO_2_, 1% O_2_) for 3 h. At the end of hypoxia, cells were treated with 50 ng/ml irisin, a dose determined in preliminary experiments and has been previously described (Jia et al., 2024). To assess the influence of AMPK and Drp1 on hypoxia-induced cellular damage, Cc (Sigma, 1 μmol/L) was coadministered during hypoxia treatment.

### Hemodynamic and cardiac functional assessments

Heparinized catheters were inserted into the left common carotid artery and connected to a PowerLab system (ADInstruments, Australia) for continuous hemodynamic monitoring. MAP was recorded every 30 min for first 2 h post-HS and hourly for an additional 6 h post-HS. To assess cardiac function, a catheter was inserted from the right carotid artery into the left ventricle, coupled with a pressure transducer to measure left ventricular parameters. Parameters included left ventricular systolic pressure (LVSP), maximum rate of pressure rise (+dP/dt_max_), and maximum rate of pressure decline (−dP/dt_max_). All parameters were recorded at matched intervals alongside MAP for 6 h post-HS.

### Histological examination

Ventricular tissue samples were fixed in 4% paraformaldehyde for 48 h. After fixation, tissues were dehydrated through a graded ethanol series and embedded in paraffin. Serial 5 μm sections were cut from paraffin blocks and stained with hematoxylin and eosin (H&E). Sections were imaged using a Nikon Eclipse CI microscope (Nikon, Japan) for morphological evaluation. Cardiac injury was histologically assessed by a pathologist blinded to the experimental groups.

### Enzyme-linked immunosorbent assay

Prior to euthanasia, approximately 6 mL of arterial blood was collected from the abdominal aorta for serum irisin quantification. Blood samples were centrifuged at 3,000 × *g* for 10 min to isolate serum. Irisin concentrations were measured using a commercial ELISA kit (E-EL-R262, Elabscience, China). Following hypoxic cell modeling, creatine kinase-MB (CK-MB) levels in cell culture supernatants were quantified using a commercial assay kit (MM-0625R2, Meimian, China). All experiments were performed in triplicate.

### Assessment of cardiac injury markers and LAC levels

Prior to euthanasia, approximately 6 ml of arterial blood was collected from the abdominal aorta to quantify cardiac troponin I (cTnI), lactate dehydrogenase (LDH), CK-MB, and LAC. Serum was isolated by centrifugation (3,000 × *g*, 10 min) and stored at −80°C. cTnI, LDH, CK-MB, and LAC levels were quantified using an automated biochemical analyzer (Mindray BS-370E, China). Cell culture supernatants were analyzed for cTnI and LDH, while CK-MB was detected using a commercial kit. All assays were repeated three times.

### Mitochondrial morphometry via transmission electron microscopy (TEM)

Following HS induction, ventricular tissue was harvested and immediately fixed in 2.5% glutaraldehyde for 2 h, incubated in 0.1 M PBS for 30 min, and post-fixed in 1% osmium tetroxide at 4°C for 2 h. Tissues were dehydrated through a graded ethanol series and embedded in resin. Ultrathin sections (60–80 nm) were cut from myocardial tissue using an ultramicrotome (UC7, Leica) and stained with uranyl acetate and lead citrate. Sections were examined using a transmission electron microscope (HT7800, Hitachi). Micrographs were analyzed at intermediate magnification using ImageJ software to quantify mitochondrial number and aspect ratio. Mitochondria were classified into three length categories: <0.6 μm, 0.6–1.0 μm, and >1.0 μm, with proportional representation calculated for each category.

### Assessment of mitochondrial function

The MMP in cardiac tissues and H9c2 cardiomyocytes was measured using a JC-1 assay kit (C2003S, Beyotime, China). JC-1-stained cardiac mitochondria were analyzed by recording fluorescence intensities at 530 nm (excitation: 490 nm) and 590 nm (excitation: 525 nm) using a microplate reader. MMP was calculated as the fluorescence intensity ratio of 590–530 nm (590/530). For cellular assays, culture medium was removed, and cells were washed with PBS, incubated with JC-1 staining solution for 1 h, and rinsed twice with JC-1 buffer. MMP changes were visualized using a fluorescence microscope.

ATP levels in cardiac tissues and H9c2 cardiomyocytes were quantified using an Enhanced ATP Detection Kit (S0027; Beyotime, China). Cardiac tissues were homogenized in ice-cold lysis buffer, centrifuged at 12,000 × g for 5 min, and the supernatant was immediately mixed with ATP detection buffer for microplate reader analysis. ATP concentrations were normalized to total protein quantified by a BCA assay kit (P0012, Beyotime, China). For cellular ATP assays, cells were washed with PBS, lysed, and centrifuged at 12,000 × *g* for 10 min. The supernatant was processed alongside tissue samples, with protein quantification performed in parallel. Experiments were repeated three times.

### Immunohistochemical analysis

Paraffin-embedded cardiac tissue sections were deparaffinized in xylene and rehydrated through graded ethanol series. Antigen retrieval was performed in citrate buffer (*pH* 6.0) at 95°C for 20 min, followed by endogenous peroxidase blocking with 3% H_2_O_2_. Sections were incubated overnight at 4°C with primary antibodies: anti-Drp1 (1:1,000, Abcam, #Ab184247) and anti-p-AMPK (1:50, Cell Signaling Technology, #2535S). After washing, species-matched HRP-conjugated secondary antibodies were applied for 1 h at room temperature. Nuclei were counterstained with hematoxylin, and immunoreactivity was developed using 3,3′-diaminobenzidine. Slides were digitized using a PRECICE 600 digital scanner (Suzhou, China) and analyzed with integrated morphometric software.

### Western blot analysis

Total protein was extracted from tissues or cells using RIPA buffer supplemented with phosphatase inhibitors (Affinity Biosciences, Wuhan, China). Protein concentrations were quantified using a bicinchoninic acid assay kit (P0010; Beyotime, China). Denatured protein aliquots (30 μg) were separated by 10% SDS-PAGE. Proteins were transferred to PVDF membranes, blocked with 5% skim milk for 1 h at room temperature. Membranes were incubated overnight at 4°C with primary antibodies diluted in 5% skim milk. After three washes with TBST, membranes were incubated for 1 h with HRP-conjugated anti-rabbit secondary antibody (1:5,000). Immunoreactive bands were visualized using a ChemiDoc™ Touch system (Bio-Rad, United States) and quantified with ImageJ software. Primary antibodies: anti-AMPK (1:1,000, Cell Signaling Technology, #2532S), anti-phosphorylated AMPK (p-AMPK; 1:1,000, CST, #2535S), anti-SIRT1 (1:1,000, Abcam, #Ab110304), anti-Drp1 (1:1,000, Abcam, #Ab184247), anti-Mfn1 (1:1,000; Abcam, #Ab221661), anti-β-actin (1:3,000, Servicebio, #GB15003-10), and anti-GAPDH (1:3,000, Servicebio, #GB15004-10).

### Reverse transcription quantitative PCR (RT‒qPCR)

Total RNA was extracted from tissue and cells using the SevenFast Total RNA Extraction Kit (Seven/Abcells, China). RNA was reverse-transcribed into cDNA using the SevenFast Two-Step RT-qPCR Kit (Seven/Abcells, China) following the manufacturer’s protocol. Primer sequences are listed in Table 1. Relative expression of Drp1, Fis1, and Mfn1 was calculated by the 2^−ΔΔCT^ method and normalized to β-actin (internal control). All experiments were performed in triplicate.

**TABLE 1:** Primer sequence.

Gene	primer F Sequence (5′–3′)	primer R Sequence (3′–5′)
Rat β-Actin	CCG​CGA​GTA​CAA​CCT​TCT​TG	GCA​GCG​ATA​TCG​TCA​TCC​AT
Rat Drp1	ACA​ACA​GGA​GAA​GAA​AAT​GGA​GT	TAC​CTT​TGG​GCA​ACA​GCT​CC
Rat Fis1	TTT​GAA​TAC​GCC​TGG​TGC​CT	TAC​CTT​TGG​GCA​ACA​GCT​CC
Rat Mfn1	CGC​CTG​TCT​GTT​TTG​GTT​GA	GCA​TTG​ACT​TCA​CTG​GTG​CA

### Statistical analysis

Data visualization was performed using GraphPad Prism 6.0 (San Diego, CA, United States). For multi-group comparisons, one-way ANOVA was conducted using SPSS 27.0 (IBM, United States), followed by Fisher’s least significant difference *post hoc* tests. Data are expressed as mean ± standard deviation (SD). Survival analysis was performed using the Kaplan–Meier method with log-rank test. Survival duration was expressed as median with interquartile range, and curves were plotted with 95% confidence intervals. Statistical significance was defined as *P* < 0.05.

## Results

### Irisin enhances survival Post-HS

**FIGURE 1:**
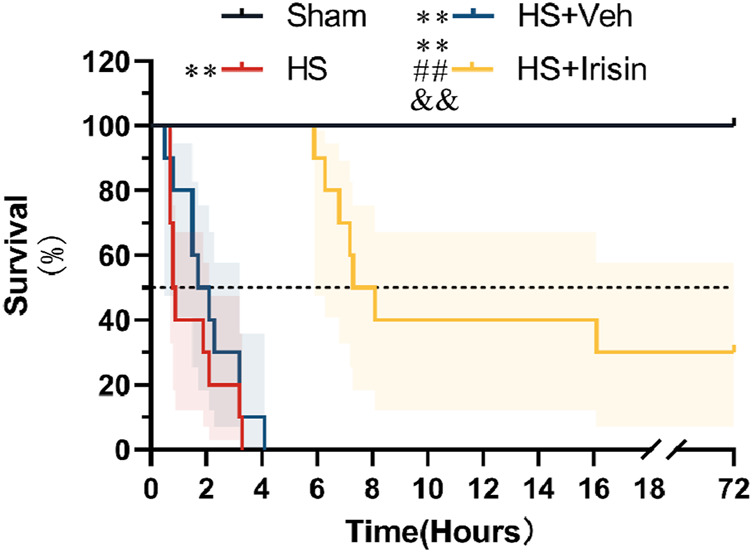
Irisin improves survival in HS. Survival analysis was performed using the Kaplan–Meier method with log-rank test. Median survival times with interquartile ranges are shown; shaded areas represent 95% confidence intervals. *p < 0.05, *p < 0.01 vs. Sham; #*p* < 0.05, ##*p* < 0.01 vs. Shock; &*p* < 0.05, &&p < 0.01 vs. HS + Veh.

Following successful induction of a lethal HS model, rats were administered exogenous irisin, and survival was monitored for 72 h. The HS group exhibited a median survival time of 0.85 h, while the Shock + Veh group showed a marginally longer median survival time of 1.9 h (*P* > 0.05, Figure 1). No significant difference in survival was detected between the HS and HS + Veh groups (*P* > 0.05, Figure 1). Notably, all animals in these groups died within 72 h, with no survivors beyond 5 h, resulting in a 100% mortality rate (Figure 1). In contrast, irisin treatment significantly increased survival rates, with 30% of animals surviving the full 72 h period and a median survival time of 7.7 h (*P* < 0.01, Figure 1). These results demonstrate that irisin significantly enhances early survival and prolongs survival duration in HS rats.

### Irisin augments MAP and ventricular function and ameliorates LAC levels

**FIGURE 2:**
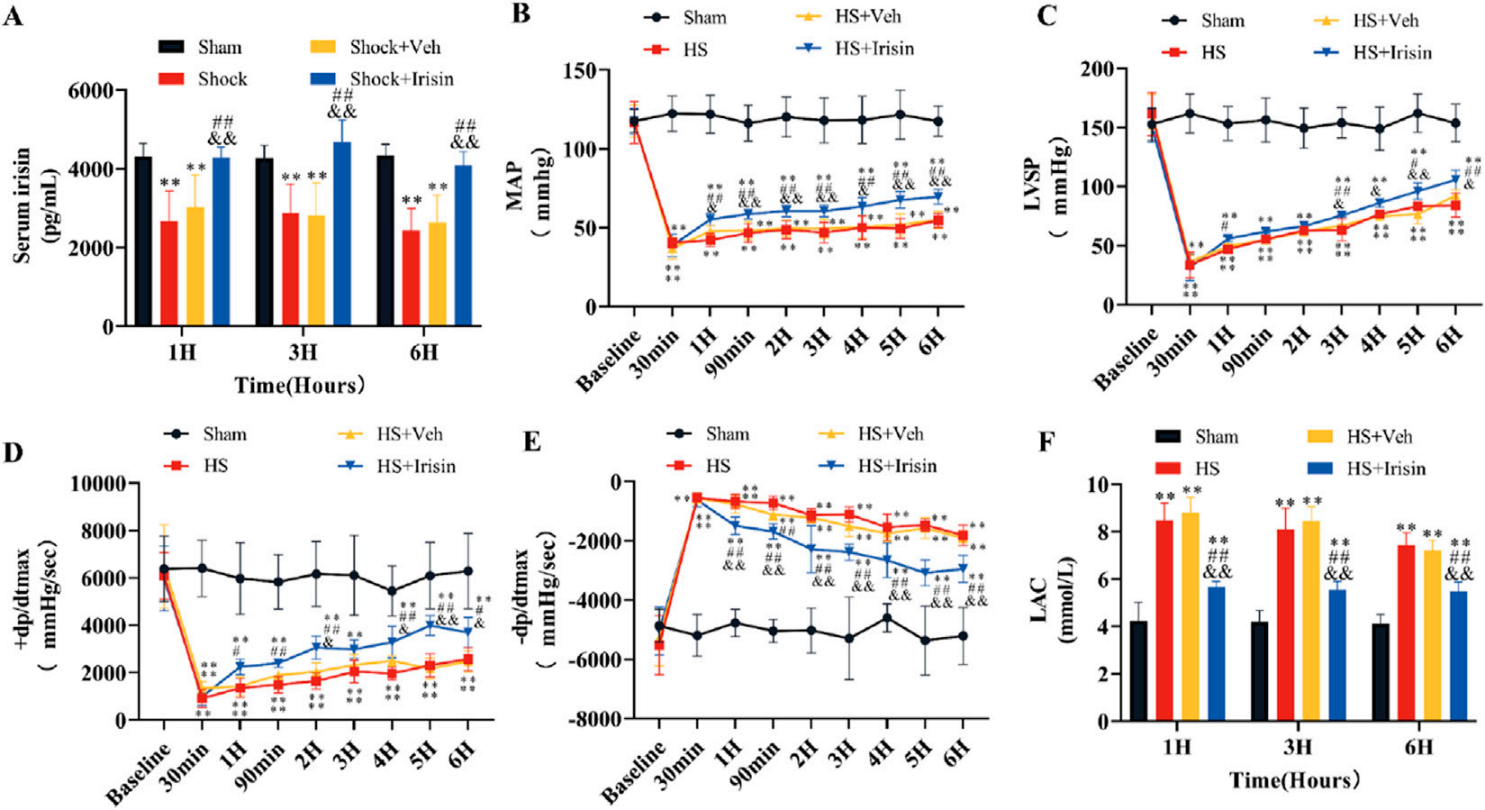
Irisin ameliorates hemodynamics and LAC levels in HS. **(A)** Serum irisin levels at 1, 3, and 6 h post-HS (n = 8). **(B)** MAP during surgery and 6 h post-HS (n = 8). **(C–E)** LVSP, +dp/dtmax, and −dp/dtmax during surgery and 6 h post-HS (n = 8). **(F)** Serum LAC levels at 1, 3, and 6 h post-HS (n = 8). Data analyzed by one-way ANOVA with Fisher’s LSD *post hoc* tests. Values expressed as mean ± SD. *p < 0.05, **p < 0.01 vs. Sham; #*p* < 0.05, ##*p* < 0.01 vs. Shock; &*p* < 0.05, &&*p* < 0.01 vs. HS + Veh.

In the HS model, serum irisin levels were markedly reduced in the HS group but improved after irisin treatment (*P* < 0.01, Figure 2A). Baseline MAP, LVSP, +dP/dt_max_, and −dP/dt_max_ showed no significant differences among the four groups before HS induction (*P* > 0.05, Figures 2B–E). Post-HS, rats displayed an initial rise in left ventricular −dP/dt_max_ alongside significant reductions in MAP, LVSP, and +dP/dt_max_. Partial recovery of these parameters occurred later through compensatory mechanisms (Figures 2B–E). However, all hemodynamic parameters remained significantly lower than Sham group levels during monitoring (*P* < 0.01, Figures 2B–E). No significant differences in hemodynamic parameters were detected between HS and HS + Veh groups at any time point (*P* > 0.05; Figures 2B–E). In contrast, irisin treatment significantly improved MAP, LVSP, +dP/dt_max_, and −dP/dt_max_ (Figures 2B–E). At 6 h post-HS, MAP increased from 54.4 ± 4.4 mmHg (HS + Veh) to 69.7 ± 4.7 mmHg in the Shock + Irisin group (*P* < 0.01; Figure 2B). Irisin administration elevated LVSP (84.2 ± 9.98 vs. 105.7 ± 8.98 mmHg, *p* < 0.05, Figure 2C), +dP/dt_max_ (2,577.8 ± 488.4 vs. 3,694.1 ± 622.5 mmHg/s; *P* < 0.05; Figure 2D), and −dP/dt_max_ (−1,812.8 ± 351.8 vs. −2,950.1 ± 454.1 mmHg/s, *P* < 0.01, Figure 2E). Irisin treatment attenuated the post-HS elevation of LAC levels observed at 1, 3, and 6 h in the HS group, though levels remained elevated compared to the Sham group (*P* < 0.01, Figure 2F). No significant difference in LAC levels was detected between HS and HS + Veh groups (*P* > 0.05, Figure 2F). These findings collectively demonstrate that irisin stabilizes cardiac function through modulation of hemodynamic recovery and LAC level.

### Irisin ameliorates cardiac damage following HS

**FIGURE 3:**
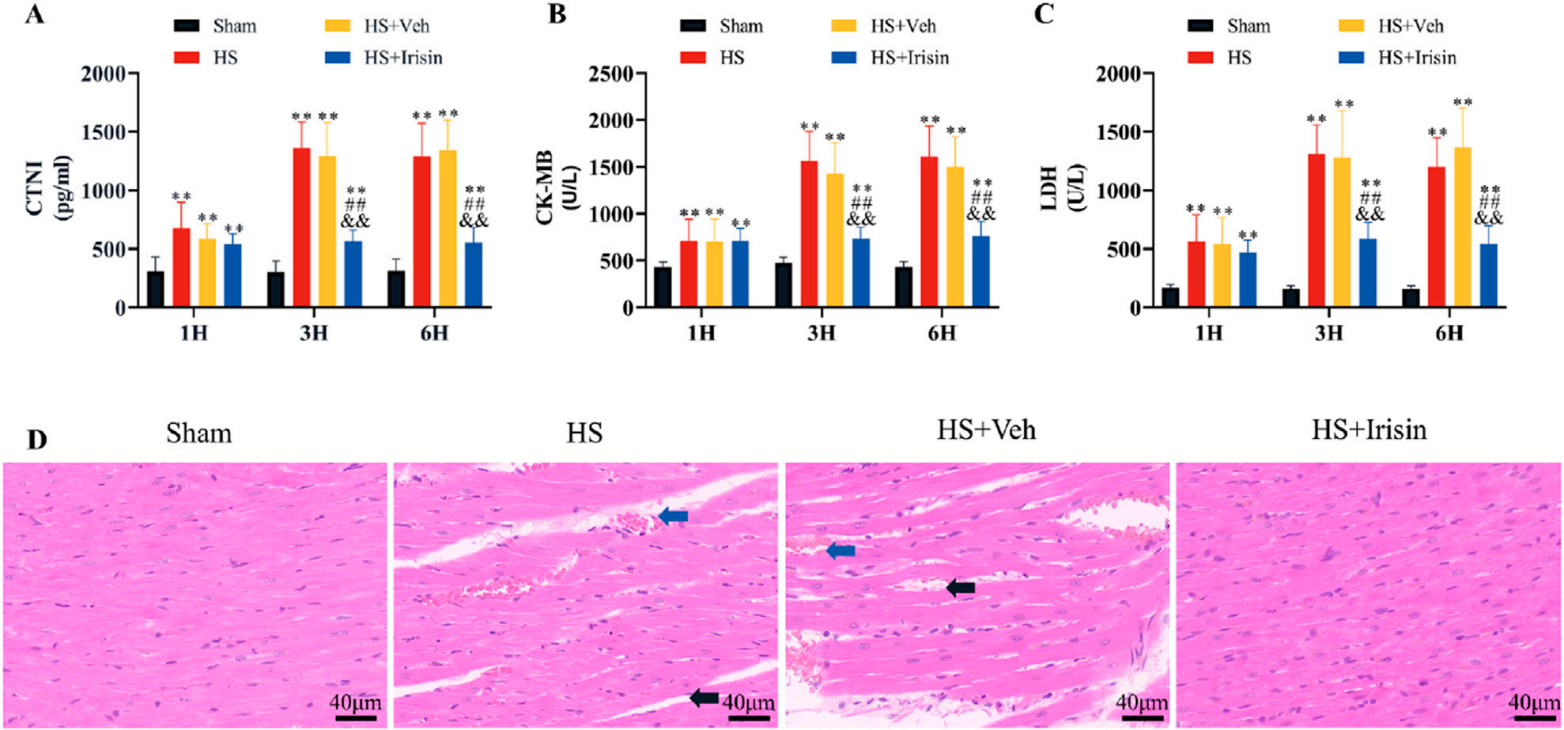
Irisin attenuates cardiac injury and preserves cardiac structure in HS. **(A–C)** Serum levels of cardiac injury markers (cTnI, CK-MB, LDH) at 1, 3, and 6 h post-HS (n = 8). **(D)** Representative H&E-stained cardiac tissue sections and quantitative analysis of pathological damage (n = 4). Data analyzed by one-way ANOVA with Fisher’s LSD *post hoc* tests. Values expressed as mean ± SD. *p < 0.05, **p < 0.01 vs. Sham; #*p* < 0.05, ##*p* < 0.01 vs. Shock; &*p* < 0.05, &&*p* < 0.01 vs. HS + Veh.

To evaluate irisin’s cardioprotective effects, serum levels of cardiac injury markers (cTnI, CK-MB, LDH) were quantified at 1, 3, and 6 h post-HS. Serum levels of cTnI, CK-MB, and LDH increased at 1 h and peaked significantly at 3 h (*P* < 0.01, Figures 3A–C). No significant differences in marker levels were observed between the HS and HS + Veh groups across all time points (*P* > 0.05, Figures 3A–C). Irisin treatment significantly reduced cardiac injury marker levels at 3 h post-HS (*P* < 0.01, Figures 3A–C). To further assess myocardial damage, histopathological analysis was performed at 3 h post-HS, coinciding with peak injury marker levels and the therapeutic window of irisin. Cardiac tissue morphology was analyzed using H&E staining at 3 h post-HS. Cardiomyocytes in the Sham group exhibited well-organized architecture, distinct myocardial fibers, and uniform intracellular spacing (Figure 3D). In contrast, the HS group displayed disrupted myocardial fiber alignment, irregular interstitial spacing, and erythrocyte extravasation (Figure 3D). Irisin treatment markedly attenuated these pathological alterations (Figure 3D).

### Irisin preserves mitochondrial function and structural integrity during HS

**FIGURE 4:**
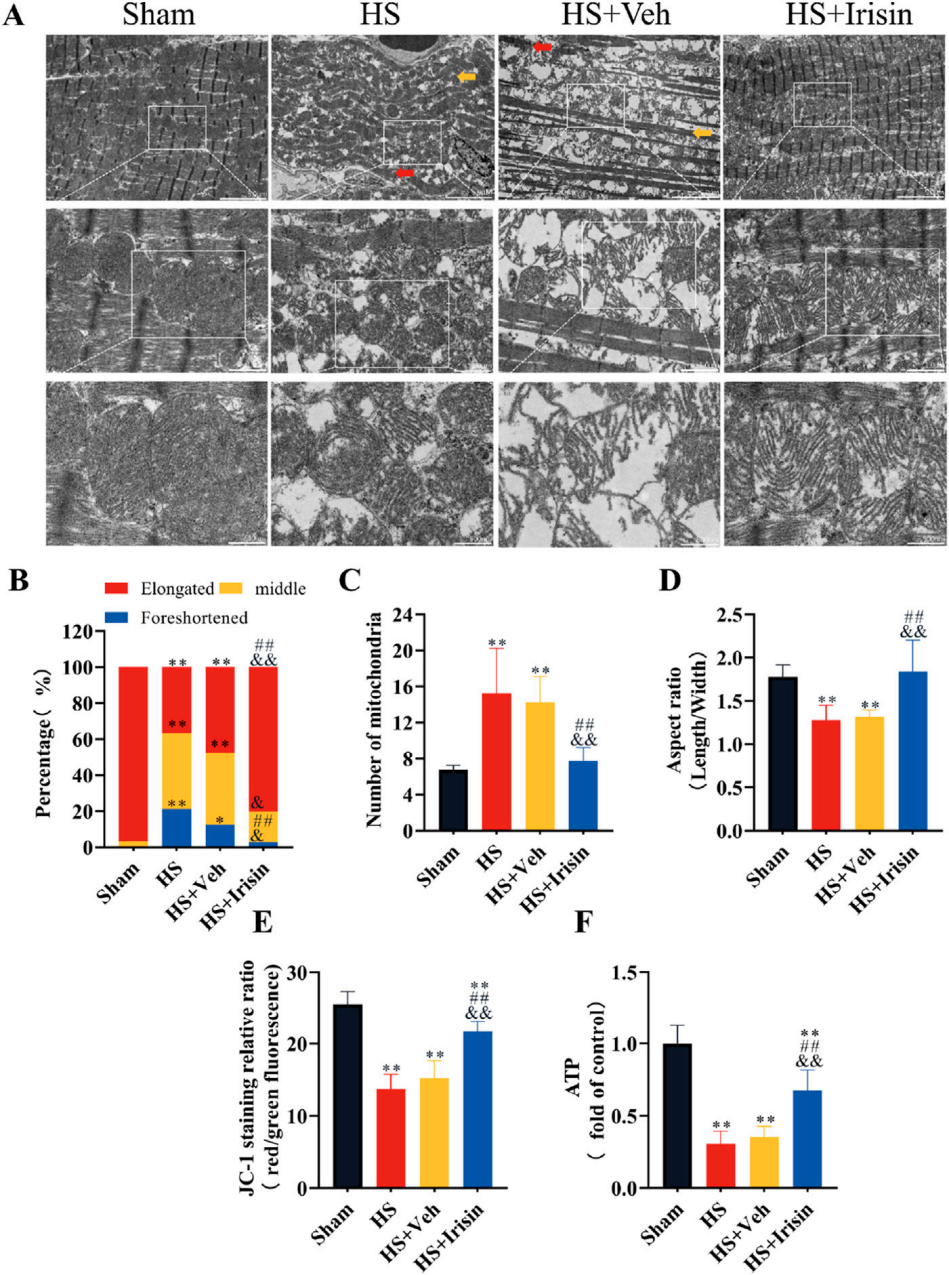
Irisin preserves mitochondrial integrity and bioenergetics in HS. **(A)** Representative TEM images of cardiac mitochondria. **(B–D)** Quantitative analysis of mitochondrial density, length, and aspect ratio (n = 4). **(E)** MMP assessed by JC-1 fluorescence ratio (red/green; n = 8). **(F)** Cardiac tissue ATP levels (n = 8). Data analyzed by one-way ANOVA with Fisher’s LSD *post hoc* tests. Values expressed as mean ± SD. *p < 0.05, **p < 0.01 vs. Sham; #*p* < 0.05, ##*p* < 0.01 vs. Shock; &*p* < 0.05, &&*p* < 0.01 vs. HS + Veh.

TEM demonstrated that cardiomyocytes in the Sham group exhibited well-organized myofibrils, distinct striations, and mitochondria with intact cristae (Figure 4A). In contrast, both HS and HS + Veh groups (which showed no intergroup differences) displayed disrupted myofibril alignment and fragmented striations in cardiomyocytes (Figure 4A). At medium magnification, TEM revealed increased mitochondrial density and reduced mitochondrial length in HS and HS + Veh groups compared to Sham group (*P* < 0.01), with no significant differences between the HS and HS + Veh groups (*P* > 0.05, Figures 4A–D). High-magnification imaging further demonstrated mitochondrial swelling and cristae disorganization in HS groups (Figure 4A). Irisin treatment restored myofibril alignment, improved striation continuity, and normalized mitochondrial architecture, characterized by elongated morphology, reduced density, and higher aspect ratio (*P* < 0.01, Figures 4A–D). These morphological alterations, suggestive of early apoptosis, prompted further investigation into mitochondrial functional changes.

Given that the MMP serves as a universal biomarker of mitochondrial function (Bazhin et al., 2020), we quantified the MMP in cardiac mitochondria post-HS (Figure 4E). Both HS and HS + Veh groups exhibited comparable MMP reduction, which was significantly attenuated by irisin treatment (*P* < 0.01, Figure 4E). No intergroup difference in MMP was observed between HS and HS + Veh (*P* > 0.05, Figure 4E). ATP levels, reflecting mitochondrial energy metabolism, were quantified to comprehensively assess functionality (Figure 4F). HS and HS + Veh groups showed equivalent ATP depletion (*P* > 0.05), while irisin treatment restored ATP levels to near-baseline levels (*P* < 0.01, Figure 4F). Collectively, irisin preserves mitochondrial structural integrity, maintains MMP, and restores ATP homeostasis during HS.

### Irisin activates AMPK and regulates mitochondrial dynamics-related factors in the heart

**FIGURE 5:**
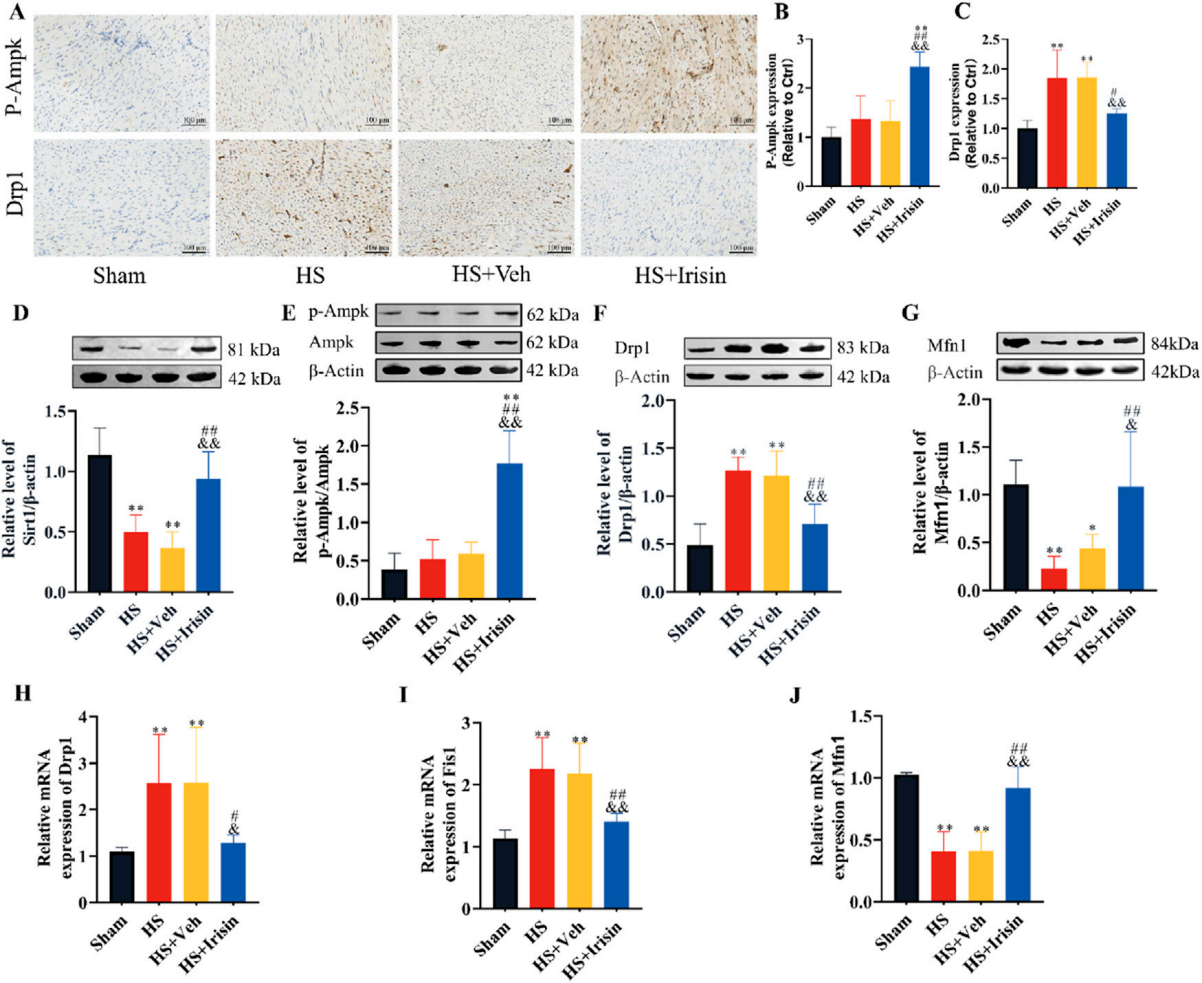
Irisin activates AMPK signaling and modulates mitochondrial dynamics in HS. **(A–C)** Immunohistochemical quantification of p-AMPK and Drp1 in rat cardiac tissues (n = 4). **(D–G)** Western blot analysis of SIRT1, p-AMPK, AMPK, Drp1, and Mfn1 protein levels (n = 4). **(H–J)** RT-qPCR analysis of Drp1, Fis1, and Mfn1 mRNA expression (n = 5). Data analyzed by one-way ANOVA with Fisher’s LSD *post hoc* tests. Values expressed as mean ± SD. *p < 0.05, **p < 0.01 vs. Sham; #*p* < 0.05, ##*p* < 0.01 vs. Shock; &*p* < 0.05, &&p < 0.01 vs. HS + Veh.

Irisin regulates metabolism, whereas SIRT1 and AMPK, functioning as the primary cellular energy sensor, critically maintain mitochondrial homeostasis (Li et al., 2019; Sánchez et al., 2022). To investigate irisin’s role in the SIRT/AMPK/Drp1 pathway during HS, we first evaluated AMPK phosphorylation and Drp1 expression via immunohistochemistry (Figures 5A–C). Post-HS, Drp1 expression was significantly upregulated (*P* < 0.01), whereas irisin treatment enhanced AMPK phosphorylation and suppressed Drp1 levels (*P* < 0.01, Figures 5A–C). Western blot analysis further confirmed that irisin significantly increased SIRT1 expression (*P* < 0.01, Figure 5D), p-AMPK (*P* < 0.01, Figure 5E), and Mfn1 expression (*P* < 0.01, Figure 5G), while downregulating Drp1 (*P* < 0.01, Figure 5F). Consistent with these findings, mRNA expression analysis of mitochondrial fusion- and fission-related genes revealed that irisin downregulated Drp1 and Fis1 and upregulated Mfn1 (*P* < 0.01, Figures 5H–J). These data demonstrate that irisin exerts cardioprotective effects during HS, likely through SIRT1/AMPK-dependent regulation of mitochondrial dynamics.

### AMPK inhibition attenuates Irisin’s protective effects against hypoxia-induced H9c2 cardiomyocytes injuries

**FIGURE 6:**
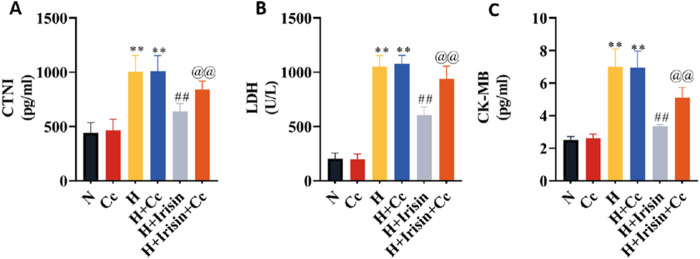
Irisin attenuates hypoxia-induced cardiomyocyte injury via AMPK-dependent mechanisms. **(A–C)** Levels of cardiomyocyte injury markers (cTnI, CK-MB, LDH) in H9c2 cardiomyocytes culture supernatants (n = 8). Data analyzed by one-way ANOVA with Fisher’s LSD *post hoc* tests. Values expressed as mean ± SD. *p < 0.05, **p < 0.01 vs. N; #*p* < 0.05, ##*p* < 0.01 vs. H; @*p* < 0.05, @@*p* < 0.01 vs. H + Irisin.

To investigate AMPK-dependent mechanisms underlying irisin’s protection in hypoxic H9c2 cardiomyocytes, we inhibited AMPK using Cc and quantified cardiac injury markers. H and H + Cc significantly increased cTnI, CK-MB, and LDH levels compared to N and N + Cc (*P* < 0.01, Figures 6A–C). Irisin treatment significantly reduced these injury markers (*P* < 0.01). However, co-treatment with Cc abolished irisin’s protective effects on hypoxia-induced injury markers (*P* < 0.01, Figures 6A–C). These findings demonstrate that irisin protects hypoxic H9c2 cardiomyocytes through AMPK signaling.

### Irisin preserves mitochondrial function in H9c2 cardiomyocytes through AMPK-mediated pathways

**FIGURE 7:**
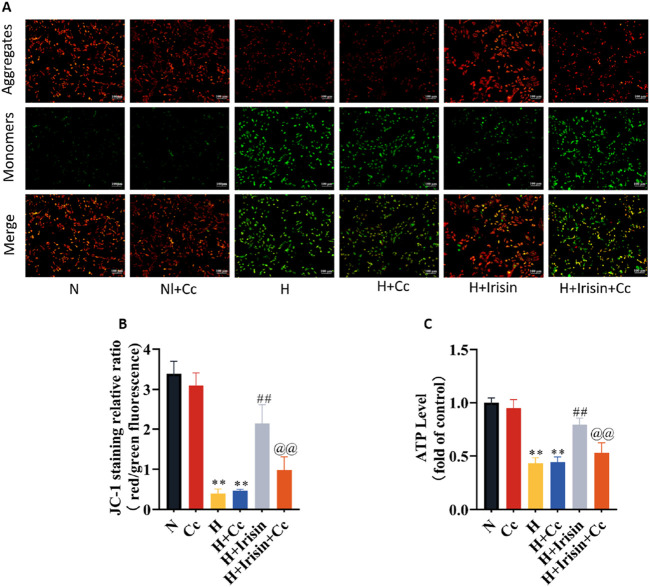
Irisin restores mitochondrial function in hypoxic H9c2 cardiomyocytes. **(A,B)** JC-1 fluorescence imaging and quantitative analysis of MMP in H9c2 cardiomyocytes (n = 4). **(C)** ATP content in H9c2 cardiomyocytes (n = 8). Data analyzed by one-way ANOVA with Fisher’s LSD *post hoc* tests. Values expressed as mean ± SD. *p < 0.05, **p < 0.01 vs. N; #*p* < 0.05, ##*p* < 0.01 vs. H; @*p* < 0.05, @@*p* < 0.01 vs. H + Irisin.

To investigate irisin’s AMPK-dependent mitochondrial protection, we observed reduced MMP and ATP levels in H and H + Cc groups versus N and N + Cc controls (P < 0.01, Figures 7A–C). Irisin treatment significantly restored MMP (P < 0.01; Figures 7A,B) and ATP levels (P < 0.01, Figure 7C). However, AMPK inhibition through Cc abolished irisin-mediated restoration of MMP and ATP in hypoxic cells (P < 0.01, Figures 7A–C). These data demonstrate that irisin preserves mitochondrial energy metabolism through AMPK-dependent mechanisms. This regulatory function highlights irisin’s potential to sustain cardiomyocyte bioenergetics during cellular stress.

### Irisin maintains mitochondrial function in H9c2 cardiomyocytes through AMPK-Dependent pathways

**FIGURE 8:**
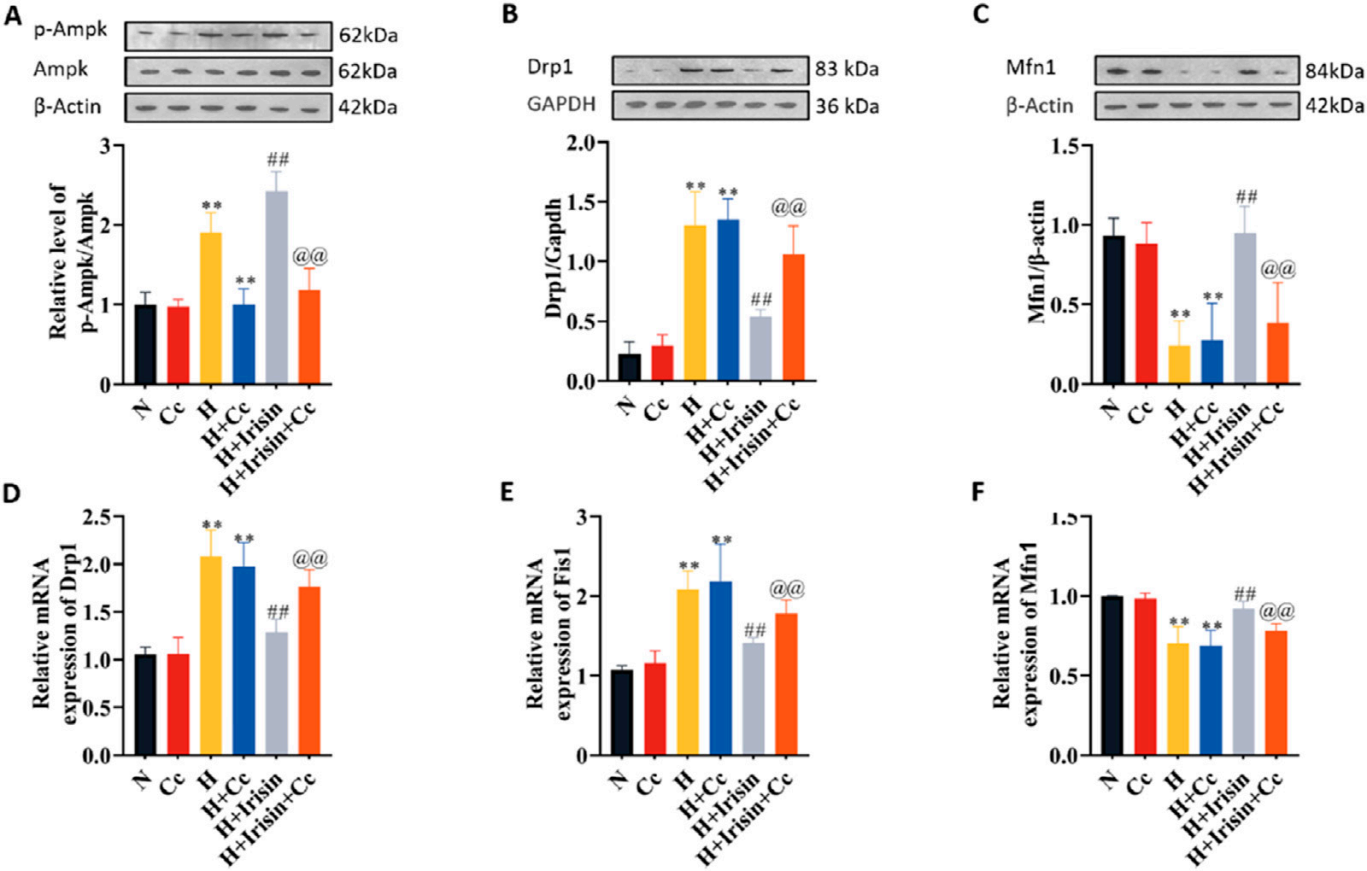
Irisin modulates mitochondrial dynamics through AMPK-ependent regulation of fission/fusion proteins. **(A–C)** Western blot analysis of p-AMPK, AMPK, Drp1, and Mfn1 protein levels in H9c2 cardiomyocytes under N, H, H + Irisin, and H + Cc (n = 4). **(D–F)** RT-qPCR analysis of Drp1, Fis1, and Mfn1 mRNA expression in H9c2 cardiomyocytes (n = 5). Data analyzed by one-way ANOVA with Fisher’s LSD *post hoc* tests. Values expressed as mean ± SD. *p < 0.05, **p < 0.01 vs. N; #*p* < 0.05, ##*p* < 0.01 vs. H; @*p* < 0.05, @@*p* < 0.01 vs. H + Irisin.

Immunoblotting assays quantified AMPK-associated proteins and mitochondrial dynamics regulators Drp1 and Mfn1. H significantly increased Drp1 expression and decreased Mfn1 levels compared to N, regardless of Cc treatment (P < 0.01, Figures 8B,C). Irisin treatment reduced Drp1 levels (P < 0.01, Figure 8B), while enhancing AMPK phosphorylation (P < 0.01, Figure 8A) and Mfn1 expression (P < 0.01, Figure 8C). Cc-mediated inhibition of p-AMPK reversed irisin’s effects on Drp1 and Mfn1 levels (P < 0.01, Figures 8B,C). Transcriptional analysis of mitochondrial dynamics genes (Drp1, Fis1, Mfn1) confirmed irisin’s *in vivo* effects: downregulating fission factors Drp1 and Fis1 (P < 0.01, Figures 8D,E) and upregulating fusion factor Mfn1 (P < 0.01, Figure 8F). Cc abolished irisin’s regulatory effects on these genes (P < 0.01, Figures 8D,E). These results demonstrate that AMPK phosphorylation is essential for irisin’s mitochondrial protection, mediated in part through Drp1 regulation. Collectively, irisin ameliorates hypoxia-induced mitochondrial dysfunction and cardiomyocyte injury via AMPK-dependent mechanisms, primarily through the AMPK/Drp1 axis.

## Discussion

This study revealed that HS induced significant reductions in serum irisin levels, which were reversed following exogenous irisin administration. Critically, irisin not only stabilized hemodynamics and attenuated cardiac injury but also markedly improved survival rates in HS rats. Mechanistically, irisin preserved mitochondrial function by modulating fission/fusion dynamics through SIRT1 - activated AMPK/Drp1 pathway, directly linking its cardioprotection to mitochondrial homeostasis. These findings establish irisin as a promising therapeutic candidate for mitigating HS-induced cardiac injury and addressing acute cardiovascular collapse.

Clinical and experimental research consistently shows that HS and subsequent resuscitation can lead to myocardial contractile dysfunction, heart failure, and cardiac injury, although the precise mechanisms underlying these effects remain to be fully delineated (Lackner et al., 2019; Tan et al., 2024). At present, therapeutic strategies for cardiac dysfunction arising from HS are predominantly supportive, with a dearth of targeted treatment options available. These findings emphasize the need for novel interventions that can specifically address the pathophysiological changes induced by HS, potentially improving outcomes in this high-risk patient population.

Irisin, secreted by skeletal muscle during physical exertion, is a bioactive peptide that exerts crucial regulatory effects on oxidative stress, inflammation, and apoptotic pathways. It is notably expressed in various organs, including skeletal muscle, cardiac tissue, adipose tissue, and renal systems (Boström et al., 2012). This peptide significantly influences metabolic processes and thermogenesis, mitigates inflammation and oxidative stress associated with hyperglycemia and obesity, and enhances energy expenditure by converting white adipose tissue to brown adipose tissue (Boström et al., 2012; Gheit et al., 2022; Behera et al., 2022; Yan et al., 2022). Irisin also has protective effects against myocardial IRI, with evidence showing its capacity to increase the MAP, heart rate, and cardiac output in zebrafish by modulating the expression of cardiovascular-related factors (Sundarrajan et al., 2017). Additionally, irisin levels in myocardial infarction patients are inversely correlated with myocardial injury markers (Abd El-Mottaleb et al., 2019), underscoring its potential in cardiac protection. Subsequent studies have verified that recombinant irisin can ameliorate cardiac function and decrease the serum levels of cTnI, CK-MB, and LDH (Wang et al., 2017; Wang et al., 2018; Yue et al., 2022). While the role of irisin in myocardial injury has been partially established, the underlying mechanisms remain elusive. To our knowledge, no study has yet definitively confirmed the protective potential of irisin against organ dysfunction induced by HS. Our research, which utilized a rat model of HS, revealed that irisin treatment markedly reduces mortality, improves MAP and LAC levels, and positively affects cardiac structure, function, and myocardial injury markers. These discoveries provide robust evidence for the protective role of irisin in cardiac injury associated with HS.

Mitochondrial preservation is of paramount importance for the amelioration of cardiac function in the early treatment of HS (Liu et al., 2024; Wu et al., 2023). Mitochondrial dysfunction is known to worsen the prognosis of HS (Zhou et al., 2024). As dynamic cellular organelles, mitochondria maintain their function through the equilibrium of their fusion and fission cycles (Giacomello et al., 2020). An overabundance of Fis1 and Drp1 proteins, coupled with decreased Mfn1 expression, can lead to excessive mitochondrial fission and a reduction in fusion. This imbalance can cause oxidative stress, disrupt the energy supply, and ultimately result in cellular death (Lei et al., 2020). Research by Wu et al. (Wu et al., 2023) demonstrated that the suppression of Drp1 expression can improve mitochondrial morphology and function, thereby extending the survival of HS rats and safeguarding vital organ functions. Sharp et al. (Sharp et al., 2014) reported that increased expression and phosphorylation of Drp1 during myocardial IRI results in mitochondrial cristae disarray, oxidative stress, mitochondrial-mediated apoptosis, and subsequent cardiac dysfunction. Our study, which showed decrease in the MMP, reduced ATP synthesis, and mitochondrial structural damage, demonstrated that cardiac functional impairment in HS is concomitant with mitochondrial dysfunction. However, irisin-based therapeutic intervention reverses these adverse changes. Irisin protects mitochondrial function and structure by decreasing Drp1 and FIS1 levels while increasing Mfn1 levels. In addition, previous studies have indicated that irisin deficiency exacerbates myocardial dysfunction and insulin resistance in diabetic mice, promotes myocardial remodelling, and intensifies hypertrophic myocardial responses induced by high-fat diets (Wang et al., 2023). These findings collectively underscore the protective role of irisin in maintaining mitochondrial integrity and function under various pathological conditions.

AMPK, a key energy sensor, interacts with SIRT1 to play a crucial and coordinative role in regulating cellular metabolism and maintaining energy homeostasis, conferring protective effects on multiple organs (Lovy et al., 2020; Trefts and Shaw, 2021; Rao et al., 2024). As upstream regulators of Drp1, AMPK and SIRT1 initially act in a Drp1-independent manner, driven mainly by the actin cytoskeleton and SIRT1-mediated deacetylation of cortactin. Later, AMPK takes the lead and orchestrates this process, which is vital for mitochondrial homeostasis (Lovy et al., 2020; Song et al., 2021; Juszczak et al., 2020; Wang et al., 2024). Studies have shown that nicotinamide activates AMPK through SIRT1-dependent mechanisms, reducing Drp1 expression and thus decreasing mitochondrial fission (Song et al., 2021). Also, AMPK activator AICAR can reduce Drp1 expression and mitochondrial fission in mice with cardiac IRI by activating AMPK (Du et al., 2022). Wang et al. (2022) reported that irisin can ameliorate vascular calcification in chronic kidney disease by activating the AMPK signalling pathway, which is associated with increased expression of the mitochondrial fission proteins Drp1 and Mfn2. In our rat model of HS, we observed an initial upregulation of Drp1 and Fis1, alongside a downregulation of SIRT1 and Mfn1. However, irisin administration upregulated p-AMPK and SIRT1 and Mfn1 and reduced Drp1 and Fis1 expression. Furthermore, Cc inhibited the activation of AMPK by irisin, thereby dampening the suppressive effect of irisin on Drp1 and Fis1 and its promoting effect on Mfn1. This led to a reversal of the protective effect of irisin on the mitochondrial and cardiomyocyte injuries induced by HS. Collectively, these findings suggest that irisin’s protective effects on mitochondrial function and the reduction in cardiac cell injury, include inhibition mitochondrial fission and the promotion mitochondrial fusion, are partly reliant on AMPK activation. These results highlight the protective role of irisin in cardiac function via the SIRT1/AMPK/Drp1 signalling pathway.

This study raises critical questions requiring future investigation. First, does irisin exhibit systemic protective effects beyond cardiac preservation in HS? Second, the long-term safety and adverse effects of irisin require further study, including dose optimization. Third, sex-specific effects of irisin in HS models warrant exploration. Fourth, whether unknown molecular mediators contribute to irisin’s effects on HS and their mechanisms requires investigation. Finally, can these mechanistic insights translate into novel therapies for traumatic HS?

Our study has limitations. First, reliance on a rodent model limits clinical translation due to the absence of human pathological samples. Second, the absence of a Sham + Irisin group precludes direct comparison of irisin’s effects under physiological versus HS conditions. Third, our study only inhibited AMPK to explore the underlying mechanisms, without examining the effects of SIRT1 inhibition. This limits our understanding of whether SIRT1 has an independent role in irisin’s protective effects beyond the AMPK/Drp1 pathway. Future studies should explore irisin’s effects across diverse physiological and pathological models, including the inhibition of SIRT1. Addressing these questions will deepen understanding of irisin’s role in HS and inform clinical translation.

## Conclusion

This study demonstrates that HS reduces serum irisin levels, whereas exogenous irisin provides multi-faceted cardioprotection. Mechanistically, irisin through SIRT1 activates the AMPK signaling pathway to suppress Drp1-mediated mitochondrial fission and enhance fusion, thereby preserving mitochondrial structural and functional integrity. This mitochondrial preservation reduces cardiomyocyte injury, improves ventricular function and MAP, and significantly reduces HS-related mortality. These findings validate irisin’s organ-protective role in HS and lay the groundwork for developing targeted therapies and clinical translation. This work also identifies critical directions for future research and therapeutic innovation in HS management.

## Data Availability

The raw data supporting the conclusions of this article will be made available by the authors, without undue reservation.
